# Auxiliary Buried‐Interface Passivation Toward Stable and Low‐Recombination‐Loss Perovskite Photovoltaics

**DOI:** 10.1002/smsc.202300218

**Published:** 2023-11-27

**Authors:** Tinghao Li, Can Wang, Chongzhu Hu, Ni Zhang, Qiu Xiong, Zilong Zhang, Feng Li, Yingyao Zhang, Jihuai Wu, Peng Gao

**Affiliations:** ^1^ CAS Key Laboratory of Design and Assembly of Functional Nanostructures Fujian Provincial Key Laboratory of Nanomaterials Fujian Institute of Research on the Structure of Matter Chinese Academy of Sciences Fuzhou Fujian 350002 China; ^2^ Fujian Normal University Fuzhou 350007 China; ^3^ Laboratory for Advanced Functional Materials Xiamen Institute of Rare Earth Materials Haixi Institute Chinese Academy of Sciences Xiamen 361021 China; ^4^ University of Chinese Academy of Sciences Beijing 100049 China; ^5^ Engineering Research Center of Environment-Friendly Functional Materials Ministry of Education Fujian Provincial Key Laboratory of Photoelectric Functional Materials Institute of Materials Physical Chemistry Huaqiao University Xiamen 361021 China

**Keywords:** auxiliary buried-interface passivations, high performances, long-term stabilities, multilayer passivations, SnO_2_, succinimide

## Abstract

Defects occur in the bulk perovskite and heterojunction interfaces of perovskite solar cells (PSCs). While additive and interface engineering are commonly used to passivate defects within the perovskite phase and at the interface, the interconnections between these two strategies have not been fully explored. This study introduces an auxiliary passivation approach for enhancing the performance of PSCs by passivating both the perovskite phase and the buried interface using succinimide (SID), referred to as the multilayer passivation (MLP) strategy. By adding SID to a lead iodide precursor solution and depositing it on tin oxide film, the remarkable ability of SID to coordinate with Pb^2+^ through Lewis‐base coordination and bind the iodide ion with hydrogen bonds is demonstrated, thereby reducing defects within the perovskite and suppressing nonradiative recombination. Additionally, SID could passivate oxygen vacancy and hydroxyl defects on the SnO^2^ surface, facilitating carrier separation and extraction. This MLP strategy enables us to achieve a power conversion efficiency (PCE) of 24.47% based on a two‐step process. Moreover, the unencapsulated devices maintain a PCE of 82% at 20 °C with 30% relative humidity after 7000 h. Overall, this study highlights the unparalleled potential of MLP strategy for enhancing the performance of PSCs.

## Graphical abstract

A novel approach to assist the regulated crystallization and passivation of perovskite interiors and buried interfaces is developed, leveraging Lewis acid–base coordination, hydrogen bonding, and electrostatic coupling using succinimide (SID) at various sites. Perovskite solar cells with improved power conversion efficiency of 24.47% for 0.1 cm^2^ and 21.90% for 1 cm^2^ are achieved.

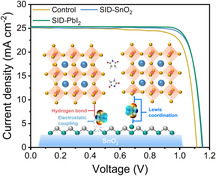

## 1 Introduction

Over the past decade, perovskite solar cells (PSCs) have made remarkable progress in photovoltaics due to their low cost, versatile manufacturing, and rapidly improving power conversion efficiencies (PCEs).^[^ ^1^, ^2^, ^3^, ^4^, ^5^, ^6^ ^]^ However, intrinsic defects within the semiconductor and unavoidable defects generated during the manufacturing process are often cited as the primary reasons that limit PSCs from approaching Shockley–Queisser limitations. For example, defects at varying energy levels can capture the electrons and holes generated by perovskite photoexcitation and result in nonradiative recombination, leading to energy loss in the transfer and conversion process.^[^ ^7^, ^8^, ^9^, ^10^ ^]^ Moreover, vacancy defects throughout the device tend to exacerbate ion migration under light, causing the continuous collapse of the perovskite structure by triggering a degrading chain reaction.^[^ ^11^, ^12^, ^13^, ^14^, ^15^ ^]^ This ultimately undermines the long‐term stability of PSCs during their storage and operation. Therefore, eliminating or passivating defects within perovskite semiconductors and at the interface with the carrier transport layer has become a significant research direction in the field of PSCs.^[^ ^16^, ^17^, ^18^, ^19^, ^20^, ^21^, ^22^ ^]^

Chemical passivation involves doping specific small organic molecules, polymers, or inorganic substances into the perovskite or transport material precursor solution, antisolvent, or coating on the surface of perovskite or carrier transport layers in various forms have been developed for eliminating the defects both in the bulk and interface.^[^ ^23^, ^24^, ^25^, ^26^, ^27^, ^28^, ^29^, ^30^, ^31^, ^32^ ^]^ This process requires that the passivating molecules reach the desired position for the target defects. For instance, depositing large organic ligands on the surface of the perovskite layer to form 2D perovskite can benefit the device's performance via combined passivation mechanisms.^[^ ^33^, ^34^, ^35^, ^36^ ^]^ Additionally, Lewis acid–base coordination was used to circumvent various undercoordinative chemical defects in perovskite.^[^ ^37^, ^38^, ^39^, ^40^ ^]^ With the rapid development of PSCs, more multifunctional passivators that can take effect on more than one type of defect via simplified procedures were intentionally developed.^[^ ^31^, ^41^, ^42^, ^43^ ^]^ For example, zwitterionic molecules with unbalanced spatial electron distribution have been found to effectively passivate defects in perovskite of different energy levels and suppress the damage caused by superoxide anions through energy passivation.^[^ ^26^, ^32^ ^]^ These attempts also feature defect passivation accompanied by other auxiliary functions, such as tuning crystallization processes. However, to meet the demand for efficient passivation, passivators with specified passivation effects on certain defects or can passivate defects from different functional layers should also be required.

These requirements led to the latest attempts to realize multilayer passivation (MLP). For example, grain regeneration and double‐sided passivation can be realized by penetrating phenethylammonium iodide (PEAI) into the buried bottom interface.^[^ ^44^ ^]^ This approach achieved omnidimensional passivation effects inside the perovskite film at the spatial level, including grain boundaries and interfaces. More than that, since the perovskite layer should be destined to contact the transport layers on both sides, for simplicity and efficiency, a passivator that can passivate both the perovskite layer and the adjacent basal layer is desirable. In one report, Xu et al. doped MDN into P3HT to create π–π stacking between P3HT and triphenylamine groups of MDN while passivating the surface defects of perovskite through the malonyl groups.^[^ ^45^ ^]^ Likewise, for perovskite precursors deposited onto the pregrown SnO_2_ electron transport layer (ETL), contact between the passivator in the precursor solution and the SnO_2_ film could be established, during which an intriguing MLP should also be expected by handling defects both inside the perovskite film and on the surface of the SnO_2_, which, however, is rarely reported.^[^ ^46^ ^]^

In this study, we investigated the effectiveness of MLP using succinimide (SID) in passivating both the perovskite layer and ETL via different procedures. As an additive in the PbI_2_ solution, the carbonyl group of SID can coordinate with Pb^2+^ to regulate the growth of perovskite crystals during the sequential deposition steps. We also observed that the hydrogen bonds between protonic N–H groups and lattice iodine could stabilize the lattice structure. Surprisingly, the SID in the pre‐deposited PbI_2_ film can reduce the density of oxygen vacancies and hydroxyl groups over the surface of SnO_2_ while enhancing electron transfer efficiency upon contact with the SnO_2_ ETL. Furthermore, we demonstrate that such crosslayer MLPs successfully improved the PCE from 22.59% for the control device to 24.47% for a small‐area target device. This improvement surpassed the PCE of cells modified with SID aqueous only on the surface of SnO_2_ film, which was 23.99%. Similarly, improved PCEs were also achieved for larger‐area devices (≈1 cm^2^), where the PCE for MLP and interface‐modified devices demonstrated a significant increase to 21.90% and 21.41%, respectively, as opposed to 20.35% for the control devices. Finally, we showed that our device maintained 93% of its initial efficiency when aged under nitrogen and 82% under 20–30% relative humidity (RH) (25 °C) over 7000 h, respectively.

## 2 Results and Discussion

**Figure 1:**
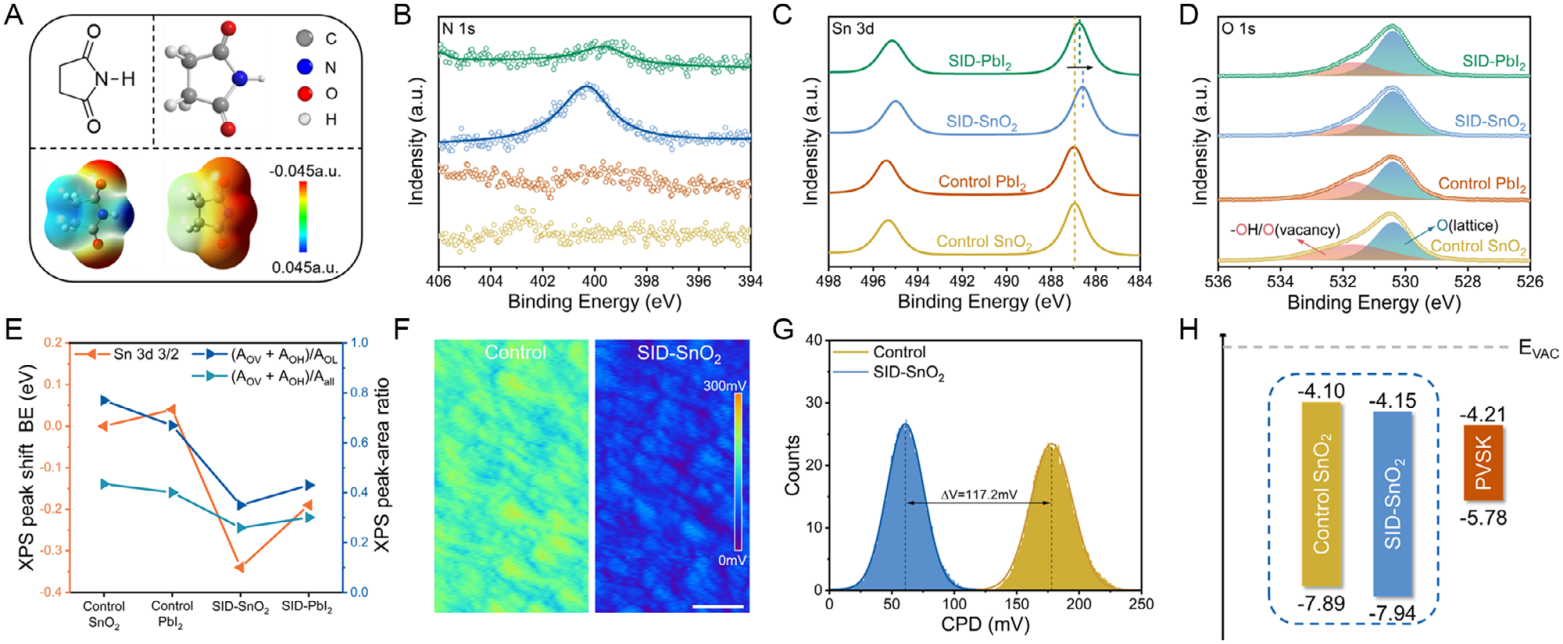
Unraveling the modified roles of SID on the surface of SnO_2_ film. A) Molecular structure and EPS of SID. The left and right sides of the lower layer correspond to the complete structure before and after losing a proton. B,C) XPS spectra of N 1*s* B), Sn 3*d* C), and O 1*s* D) peaks of pristine, pure PbI_2_ modified, SID aqueous solution modified, and SID–PbI_2_‐modified SnO_2_ films. The blue and pink peaks in (D) correspond to lattice oxygen and oxygen vacancy or hydroxyl oxygen, respectively. E) XPS peak displacement (the orange triangle pointing to left) and XPS peak area ratio (the blue triangle pointing to right) extracted from the XPS spectral fitting curve. F) KPFM and G) surface CPD distributions of SnO_2_ films with and without SID modification. H) The energy‐level diagrams of various ETLs and control PVSK.

**Figure** **1**A displays the molecular structure and simulated electrostatic potential images of SID, illustrating the charge distribution of the molecule in a 3D way. With its cyclic imide structure, SID features a conjugated system that combines the nitrogen atom with two carbonyl groups. The lone pair of electrons on the nitrogen is conjugated to the carbonyl (C═O), resulting in a partial transfer of the lone pair to the oxygen, bringing the electrons on the N–H bond closer to the nitrogen atom and making the hydrogen more prone to depart as a proton. As a result, SID manifests a strong dipole moment.

In order to comprehensively evaluate and fully exploit the passivation effect of SID on various defect sites, we carried out two different treatments: 1) spin coating an aqueous SID solution onto the SnO_2_ film (Figure S1A, Supporting Information) and 2) incorporating SID molecule with PbI_2_ (Figure S1B, Supporting Information), which we referred to as SID–SnO_2_ and SID–PbI_2_ (or SIID–PVSK) devices, respectively. Then, we carefully compared the simultaneous passivation effects of these two treatments on SnO_2_ layer and perovskite layer.

### 2.1 SID Passivation Effect on Hydroxyl and Oxygen Vacancies of SnO_2_ Layer

The electronic interactions of SID with SnO_2_ were detected by X‐Ray photoelectron spectroscopy (XPS) measurements under different treatments. Note that all films containing PbI_2_ were rinsed off with DMSO solvent after annealing to remove the influence of Pb^2+^ (Figure S2, Supporting Information). As shown in Figure 1B, an N 1*s* peak with an *E*_b_ of 400.6 eV appeared in the SnO_2_ film that underwent surface modification with SID aqueous solution, while no such peak was observed in the pristine SnO_2_ film, indicating the successful coating of SID on the surface of the SnO_2_ film. Furthermore, the N 1*s* peak was still discernible in the exposed SnO_2_ film after rinsing off the annealed SID–PbI_2_ film, although its intensity was lower than that of the SnO_2_ film modified with SID aqueous solution. In contrast, no N 1*s* peak could be found on the SnO_2_ film modified with pure PbI_2_ solution.

Furthermore, the E_b_ of the Sn 3*d* 5/2 (486.91 eV) and Sn 3*d* 3/2 (495.33 eV) peaks of the SnO_2_ film decreased to 486.57 and 494.98 eV, respectively, after SID aqueous solution modification (Figure 1C). The identical downshifting of 0.35 eV toward a lower electron *E*_b_, indicates that the chemical environment around Sn^4+^ was altered in the modified SnO_2_, possibly due to charge compensation between C=O and uncoordinated Sn^4+^. This compensation resulted in a slight increase in the density of the electron cloud around Sn^4+^, with its valence state likely lowered by SID. Additionally, differences in the peak positions were observed after washing the two different PbI_2_ films, and we have summarized all the peak displacement information to facilitate observation, as shown in Figure 1E. Compared to pristine SnO_2_, the two peaks of the pure PbI_2_‐covered film shifted by 0.04 eV toward higher *E*_b_. In contrast, the SID–PbI_2_‐covered film shifted down by 0.19 eV toward a lower *E*_b_, which is consistent with the change in *E*_b_ from the films after modification by SID aqueous solution, but not to the same extent of signal displacement. These results suggest the strong chemical interactions between the Sn^4+^ of the SnO_2_ film and the carbonyls of the SID, even though SID is used as additive in PbI_2_ solution. These interactions provide the favorable conditions for SID to achieve MLP.

To investigate the underlying mechanism of SID passivation of the SnO_2_ film surface, we analyzed the O 1s spectra. Figure 1D shows us an asymmetric spectrum profile for both pristine and treated SnO_2_ films. The asymmetric peaks of each sample can be deconvoluted into two peaks with different binding energies, corresponding to two distinct oxygen species. The peak at ≈530.6 ± 0.06 eV is attributed to the saturated lattice oxygen (O_L_) in the SnO_2_ film, while the other peak at 531.8 ± 0.06 eV represents the intrinsic oxygen vacancy (O_V_) and the chemisorbed oxygen atoms or hydroxyl groups (O_OH_).^[^ ^47^ ^]^ Notably, after two different SID treatments, the intensity of lattice oxygen increased, and the intensity of adsorbed oxygen reduced. The peak area ratio (*A*_OV_ + *A*_OH_)/A_all_, where *A*_OV_, *A*_OH_, and *A*_all_ represent the peak area of O_V_, O_OH_, and all the O1s peaks, respectively, can be used to determine the relative content of oxygen vacancy and hydroxyl group in SnO_2_ films. In this study, the peak area ratio decreased from 0.44 (pristine SnO_2_) to 0.26 (SID‐modified SnO_2_), indicating the effective reduction of hydroxyl and oxygen vacancy on the SnO_2_ surface modified by SID aqueous solution. This may result from the SID Lewis acid property (*pK*_a_ = 9.62), whose proton could react with hydroxyl groups on SnO_2_ film (Figure S3, Supporting Information). In contrast, the SnO_2_ film covered by pure PbI_2_ solution showed a minor decrease to 0.40. Further, the SnO_2_ film covered by SID–PbI_2_ showed a significantly reduced peak area ratio of 0.30, which is still higher than that of the SID‐modified SnO_2_ film. This result could be attributed to the fact that the passivating ability of SID–PbI_2_ on the hydroxyl group defect is weaker than SID aqueous solution.

To investigate the effect of two different SID treatments on the morphology of SnO_2_ films, we conducted scanning electron microscopy (SEM). No significant difference was observed between the SID–SnO_2_/SID–PbI_2_ and control films (Figure S4, Supporting Information). However, atomic force microscopy analysis of relevant samples revealed slightly reduced root‐mean‐square (RMS) roughness of the SID–SnO_2_ films to RMS = 24.82 nm from RMS = 25.22 nm for the control films. This reduction may be conducive to depositing perovskite films with large grains and few grain boundaries (Figure S5, Supporting Information). Additionally, Kelvin probe force microscopy (KPFM) shows that the SID modification results in a lower contact potential difference (CPD) and uniform potential distribution (Figure 1F–G). The calculated Fermi levels are shown in Figure S6, Supporting Information. Furthermore, the favorable energy band alignment between the perovskite and SnO_2_ surfaces can prevent nonradiative recombination caused by the accumulation of photogenerated electrons at the interface, thereby reducing recombination loss and device instability (Figure 1H and S7, Supporting Information).

### 2.2 SID Passivation Effect on Uncoordinated Pb^2+^ and Hydrogen Bonds with I of Perovskite Layer

**Figure 2:**
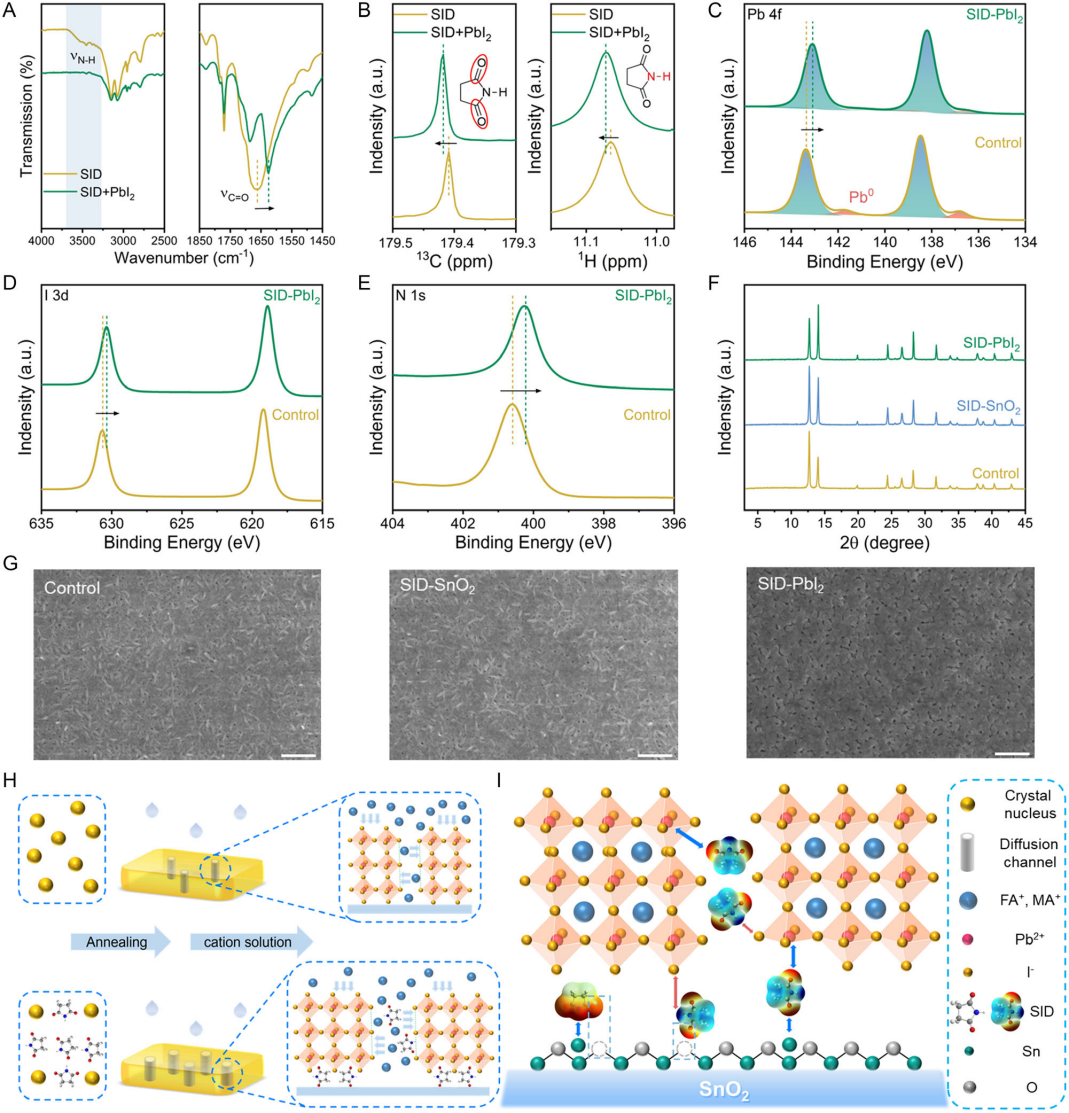
Characterizations of perovskite/SID chemical interaction. A) The FTIR spectra for SID powder and SID + PbI_2_ powder. B) ^13^C NMR spectra and ^1^H NMR spectra of SID or SID + PbI_2_ in DMSO‐d6 solution. C–E) XPS spectra of C) Pb 4*f* , D) I 3*d*, and E) N 1*s* peaks of control and SID–PbI_2_‐based perovskite films. F) XRD patterns of perovskite films. G) Surface SEM images of control PbI_2_ film deposited on pristine or SID–SnO_2_ substrate and SID–PbI_2_ film. The scale bar is 1 μm. H–I) Schematic illustration of a chemical mechanism for regulating the crystallization of lead iodide films and passivating defects using SID.

The interaction between SID and PbI_2_ can be preliminarily verified by comparing the Fourier‐transform infrared (FTIR) spectroscopy of SID and its mixtures with PbI_2_ (**Figure** **2**A and S8, Supporting Information). Compared with pure SID, it can be distinctly observed that the N—H bond stretching vibration peak at 3300–3500 cm^−1^ becomes weaker and broader in the mixture, which may represent that the N—H bond strength is weakened due to the formation of hydrogen bond between N—H bond and iodide ions. In addition, the two stretching vibration peaks of C=O at 1707 and 1662 cm^−1^ become more pronounced and migrate toward the lower frequency to 1687 and 1625 cm^−1^ due to the electrostatic interaction between the positively charged Pb^2+^ and the electron‐rich carbonyl group. The strong electron‐absorbing induction effect of Pb^2+^ causes the electron cloud on the carbonyl group to shift toward the oxygen atom, weakening the electron cloud density in the middle of the C=O bond and the force constant, which causes the absorption to shift in the direction of the lower wavenumber.^[^ ^28^ ^]^ The electron delocalization of the N–H and C=O groups represents the formation of hydrogen bonds and Lewis acid–base adduction between PbI_2_ and SID, demonstrating a strong interaction.

In order to obtain a further understanding of the chemical interaction between PbI_2_ and SID at the atomic level, we measured the nuclear magnetic resonance (NMR) spectroscopy for SID and its mixtures with PbI_2_, which were dissolved in deuterated dimethylsulfoxide (DMSO‐d^6^) (Figure 2B and S9, Supporting Information). Changes in chemical shifts in NMR spectra are indications of the changed chemical environment of the nucleus. As shown in Figure 2B, the peak of ^13^C NMR from C=O in the pure SID was shifted slightly toward the lower field in the mixture sample, indicating the electron density change of carbonyl groups. Meanwhile, strong electron conjugation results in two ^1^H NMR peaks at 11.065 and 3.354 ppm assigned to N–H and α–H, respectively, shifting toward the lower field to 11.072 and 3.359 ppm after adding PbI_2_. The formation of hydrogen bonds reduced the electron cloud density around the hydrogen, thus weakening the shielding effect of electrons, leading to the downward chemical shifts to lower fields.^[^ ^48^, ^49^ ^]^

We conducted further characterizations to better understand the changes in the perovskite. XPS was used to study the surface chemistry of the perovskite films by accurately measuring the inner electron binding energy (*E*_b_) of the atoms (Figure 2C–E and S10, Supporting Information). The C 1*s* spectrum was used to calibrate all XPS spectra. The control film showed two prominent peaks at 138.48 and 143.35 eV, corresponding to Pb 4*f* 7/2 and Pb 4*f* 5/2, respectively. In contrast, the SID–PbI_2_ film exhibited two main peaks at 138.23 and 143.10 eV, both shifted by 0.25 eV toward the lower electron *E*_b_ (Figure 2C). This shift is attributed to the carbonyl group replenishing the Pb^2+^ nucleus with an additional electron cloud through Lewis acid–base interactions, causing the Pb nucleus to use some of the Coulomb forces to maintain the extra electron cloud. As a result, the binding of the inner electron is weakened, which is manifested as the peak movement of Pb 4*f* toward the lower *E*_b_.

Furthermore, the two shoulder peaks located at 138.48 and 143.38 eV correspond to Pb^0^, a deep‐level defect commonly found on the surface of perovskite. Pb^0^ is the degradation product of residual PbI_2_ in the process of device preparation under light or X‐Ray irradiation. The formation of Pb^0^ in both PbI_2_ and perovskite generates new nonradiative recombination centers, suppressing the generation and transfer efficiency of charge carriers and ultimately affecting the PCE and stability of PSCs.^[^ ^50^ ^]^ It was observed that Pb^0^ almost disappeared after the addition of SID, indicating that the electrostatic coupling between SID and Pb^2+^ effectively suppressed the reduction of Pb^2+^ and passivated the surface defects of perovskite. Additionally, as shown in Figure 2D–E, the films passivated by SID displayed downward shifted binding energies of I and N than those in the control film, with a shift of 0.28 eV for I and 0.37 eV for N. This shift could be attributed to the formation of hydrogen bonds between N–H and I, confirming effective electron transfer between the passivator and perovskite simultaneously. The N 1*s* spectra cannot be deconvoluted into the MA signal, which may result from the substation of MA ion by FA ion in the cationic site.

### 2.3 Crystalline and Morphology of Perovskite Films

To evaluate the distribution of the passivator at different depths within the perovskite film, we acquired energy‐dispersive spectrometer mapping images (Figure S11 and S12, Supporting Information). As expected, in the sample with SID–SnO_2_ film, more C elements were detected at the bottom of the perovskite film, demonstrating the enrichment of SID at the contact with the SnO_2_ ETL. X‐Ray diffraction (XRD) measurements were performed to investigate the effect of SID–SnO_2_ and SID–PbI_2_ on the crystalline structure of perovskite thin films. As shown in Figure 2F, all types of perovskite films show analogous cubic perovskite structures. Compared to the control perovskite films, the SID‐modified films demonstrated amplified diffraction intensity and reduced half‐width at half‐maximum, indicating improved vertical orientation and crystallinity. Additionally, all films showed relatively strong diffraction peaks of PbI_2_ due to the two‐step process. Nevertheless, the diffraction intensity of PbI_2_ peaks decreased significantly after SID addition, demonstrating the strong capability of SID to combine with PbI_2_. To visualize the crystallinity enhancement, we obtained the films depositing only PbI_2_ and found that both the SID–SnO_2_ and control SnO_2_ substrate showed aciculate PbI_2_ crystals (Figure 2G). However, SID–PbI_2_ films exhibited a more mesoporous structure, which could be attributed to the low nucleation center density induced by the interaction between SID and the Pb–I framework.^[^ ^51^ ^]^ The increased contact area of the PbI_2_ film has been shown to enhance the mosaic efficiency of organic cations in the Pb–I lattice. This phenomenon promotes the crystal growth of perovskite films, reducing interfacial defects caused by residual PbI_2_ from the precursor.^[^ ^52^ ^]^ Subsequent XRD measurements of PbI_2_ film revealed that the SID–PbI_2_ film demonstrated significantly enhanced crystallinity, indicating that the addition of SID effectively regulates PbI_2_ crystallization, laying the foundation for high‐quality perovskite films in the following steps (Figure S13, Supporting Information).^[^ ^53^ ^]^

Adding SID in perovskite films results in a more uniform and pinhole‐free compact morphology with a larger average grain size (Figure S14, Supporting Information), consistent with XRD results. Cross‐sectional sweep electron micrographs indicate that the thickness of the perovskite layer is 714 nm for the control device, 775 nm for the SID–SnO_2_ device, and 778 nm for the SID–PbI_2_ device (Figure S15, Supporting Information). The perovskite films with SID feature penetrating grains and thicker perovskite films, which could enhance light collection efficiency and short‐circuit current of devices. Additionally, the RMS of 32.93 nm is lower than the RMS of 33.59 nm observed for the control film (Figure S16, Supporting Information), consistent with the smooth morphology exhibited by SEM images. This lower roughness could reduce the interfacial holes between the hole transport layer and perovskite, effectively avoiding carrier transport loss. Furthermore, KPFM indicated that SID–PbI_2_ films exhibited a higher CPD and uniform potential distribution, which is conducive to better charge transmission (Figure S17, Supporting Information).

Based on the above analysis, we proposed the chemical mechanism for regulating crystallization and passivating defects using SID as shown in Figure 2H–I. The high electron cloud density of the carbonyl groups in SID facilitates its chelation with uncoordinated lead in the Pb–I framework, inhibiting the accumulation of free PbI_2_. This interaction also creates low‐density nucleation centers that facilitate the crystal growth of PbI_2_ films with higher crystallinity and a more porous mesoporous structure. This growth process enables the efficient embedding of organic cations into the PbI_2_ lattice, preventing excessive residue from forming high‐quality perovskite films (Figure 2H).^[^ ^48^ ^]^ As SID is evenly distributed in the perovskite layer and on the SnO_2_ surface, the positively charged N–H group provides hydrogen bonds with lattice iodine, stabilizing the crystal structure and impeding ion migration. Furthermore, the relatively facile removal of the proton from the amine also provides the possibility to react away the hydroxyl groups on the surface of SnO_2_ film.^[^ ^20^ ^]^ After proton removal, the conjugated structure exhibits an augmented electron cloud density, thereby enhancing the coupling with undercoordinated Sn^4+^, limiting reduction after oxygen extrusion and improving electron transport efficiency (Figure 2I).

### 2.4 Nonradiative Recombination and Charge Transfer Dynamics

**Figure 3:**
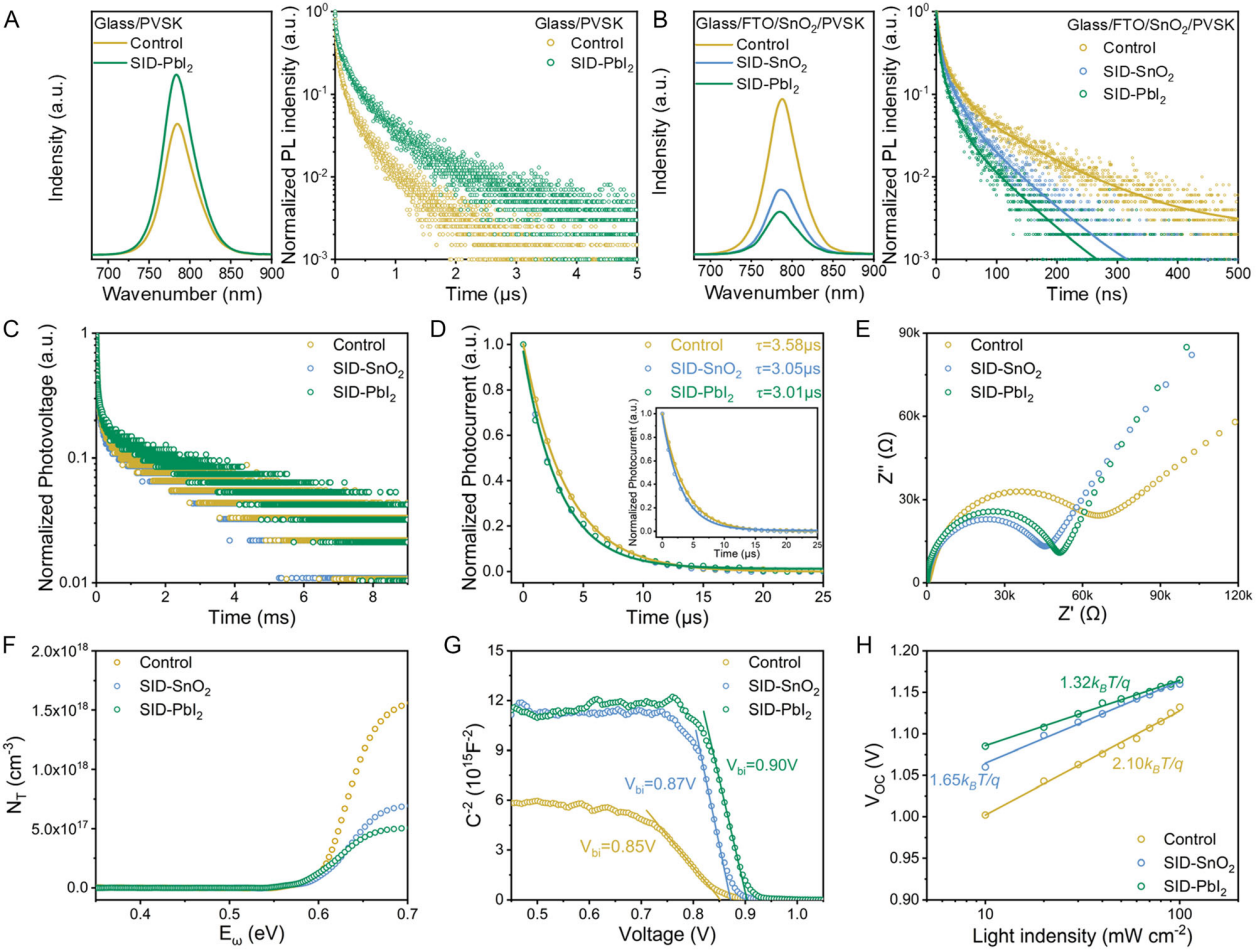
A) PL and TRPL spectra of perovskite coated on glass. B) PL and TRPL spectra of perovskite with the ETL characterizations of carrier dynamics and trap density. C) TPV decay, D) TPC decay, E) the Nyquist plots, F) calculated trap density (N_T_), G) Mott–Schottky plots, and H) relationship between *V*_OC_ and the light intensity for the control, SID–SnO_2,_ and SID–PbI_2_ PSCs.

To investigate the defects of perovskite films, we explored two half devices with and without SID modification through steady‐state photoluminescence (PL) and time‐resolved photoluminescence (TRPL) spectroscopy. When photoexcitation occurs in perovskite films deposited on glass without an ETL, the electrons inside the perovskite transition to the permitted excited state. When these excited electrons return to the thermal equilibrium state, they typically emit light or are captured by defects at the middle level through nonradiative recombination to release energy. Therefore, the luminous intensity is usually inversely proportional to the density of the defect state inside the film. As shown in **Figure** **3**A, the PL strength of SID–PbI_2_ films increases significantly, indicating effective passivation of nonradiative recombination centers in the perovskite layers by SID. Furthermore, as confirmed by TRPL spectra, the average fluorescence of the carriers in the modified films increases from 201 to 361 ns, which is consistent with the PL spectra (Table S1, Supporting Information). This prolonged carrier lifetime implies a longer carrier diffusion length, indicating that SID incorporation can significantly reduce the defect‐assisted recombination rate.^[^ ^54^ ^]^ Ultraviolet–visible absorption measurements (Figure S18, Supporting Information) show that the spectra of the treated perovskite and control films were nearly identical, demonstrating that the addition of SID has little impact on the composition and absorption properties of perovskite.

PL and TRPL spectra obtained from the backside of the device can provide information on the charge carrier dynamics at the interface between perovskite and SnO_2_. We collected PL and TRPL spectra for three architectures: SnO_2_/perovskite, SnO_2_/SID/perovskite, and SnO_2_/SID‐doped perovskite. Both devices with SID exhibited faster photoluminescence decay rates (Figure 3B, and Table S1, Supporting Information), with the SID–PbI_2_ film showing faster initial decay than the SID–SnO_2_ films. This indicates a faster charge extraction and transfer process at the interfaces of both modified samples, but the SID directly treated interface showed better passivation efficiency.^[^ ^55^ ^]^

We further investigated carrier dynamics and defect density in control and target devices through electrochemical characterization, which are essential physical factors affecting device performance. Transient photocurrent (TPC) and transient photovoltage (TPV) decay processes were applied to study the carrier behavior in PSCs, with TPC used to exhibit carrier density and transmission processes and TPV used to exhibit carrier lifetime and recombination processes. As depicted in Figure 3C, the photovoltage decay of SID–SnO_2_ films and SID–PbI_2_ films decreased sequentially compared to the control films, indicating an increase in carrier diffusion length, implying that SID effectively passivates defects resulting in fewer trap states in the device. Furthermore, we observed extraction lifetime of 3.58, 3.05, and 3.01 ms for the control, SID–SnO_2_, and SID–PbI_2_ films, respectively, indicating a significant improvement in carrier extraction (Figure 3D). The addition of SID could passivate defects inside and at the interface of perovskite, optimize the interfacial contact between the perovskite layer and ETL, and thus promote the transmission of photoexcited carriers. These results align with the trends observed in PL and TRPL.

Moreover, the SID–PbI_2_ samples demonstrated the optimal charge transfer and recombination constant, suggesting that doping SID into the PbI_2_ films can effectively suppress charge recombination and promote charge transfer. To further characterize the carrier transport and recombination behavior, we utilized electrochemical impedance spectroscopy (EIS). Figure 3E shows the Nyquist plots of the corresponding devices measured by an electrochemical workstation in the dark. The low‐ and high‐frequency semicircles are assigned to the interfacial charge transport resistance (*R*_ct_) and the interfacial recombination resistance (*R*_rec_), respectively. Compared with the control devices, the *R*_ct_ of SID–SnO_2_ and SID–PbI_2_ devices sequentially decreased while *R*_rec_ increased (refer to Table S2, Supporting Information). Therefore, it can be speculated that the SID modification suppresses charge recombination at the interface and improves carrier extraction efficiency.

To quantify the carrier mobility of the two SID‐treated devices, we prepared electron‐only devices with the structure of FTO/SnO_2_/perovskite/PCBM/Au with three types of perovskite layers and tested them for space–charge‐limited currents (SCLC). In the trap‐free SCLC region, carrier mobility can be calculated according to Mott–Gurney's equation.

(1) $$J_{D}=\frac{9\mu \varepsilon_{r}\varepsilon_{0}V_{b}^{2}}{8L^{3}}$$

where *μ* is mobility, *ε*_r_ is the relative dielectric constant, *ε*_*0*_ is the vacuum permittivity, *V*_b_ is the bias voltage, and *L* is the perovskite film thickness.^[^ ^56^ ^]^ The electron mobility of the control, SID–SnO_2_, and SID–PbI_2_ devices calculated from SCLC was 1.63, 4.84, and 4.20 cm^2^V^−1^ s^−1^, respectively (Figure S19A–C, Supporting Information). Consistent with the TPC and EIS measurements, better interfacial contact after SID modification benefited the charge transfer efficiency. Typically, interfacial modification is known to improve carrier mobility, but we found that various tests exhibited significantly better carrier mobility for SID–PbI_2_ devices than for SID–SnO_2_ devices. This could be due to the less effective interfacial passivation of SID–PbI_2_ compared to that of SID‐SnO_2_. Nevertheless, doping SID into perovskite can produce highly crystalline perovskite films, implying fewer non‐radiative recombination centers, high carrier density, and unobstructed carrier transport channels inside perovskite films, which further improve carrier transport. In addition to carrier mobility, SCLC is more commonly used to quantitatively measure the trap density (*N*_T_) within perovskite films.^[^ ^57^ ^]^ The current exhibits a linear Ohmic response in the low‐bias region, reflecting the Ohmic device response, and a rapid nonlinear growth with increasing bias in the high‐bias region, indicating a defect‐filling process. N_T_ can be calculated from the following equation.

(2) $$N_{T}=\frac{2V_{\text{TEL}}\varepsilon_{r}\varepsilon_{0}}{qL^{2}}$$

where *V*_TFL_ is the trap‐filling voltage and *q* is the elementary charge. The defect density of the SID‐PbI_2_ film decreased significantly to 1.43 × 10^15^ cm^−3^, which was significantly lower than those of control film (2.98 × 10^15^ cm^−3^) and SID–SnO_2_ film (1.60 × 10^15^ cm^−3^).

The energy distribution of defects in perovskite films was further characterized by means of thermal admittance spectroscopy.^[^ ^16^ ^]^ The defect density was determined from the frequency–capacitance relationship, which was modeled through the following equation.

(3) $$N_{T}\left(E\omega \right)=-\frac{V_{\text{bi}}}{qW}\frac{dC}{d\omega }\frac{\omega }{k_{B}T}$$

where *V*_bi_ is the built‐in potential, *W* is the depletion width, *C* is the capacitance, *ω* is the angular frequency, *k*_B_ is the Boltzmann constant, and *T* is the temperature. The corresponding energetic demarcation (*E*_ω_) could be defined with ω according to the following function.

(4) $$E_{\omega }=k_{B}T\text{ln}\left(\frac{\omega_{0}}{\omega }\right)$$

where *ω*_*0*_ is the attempt‐to‐escape frequency, which could be calculated with the capacitance map from the frequency dependence through the relaxation process (Figure S20, Supporting Information).^[^ ^58^, ^59^ ^]^ As shown in Figure 3F, the density of deep‐level trap states in both SID–SnO_2_ and SID–PbI_2_ devices is significantly reduced to 6.93 × 10^17^ and 5.03 × 10^17^ cm^−3^, respectively. This is an order of magnitude decrease compared to 1.53 × 10^18^ cm^−3^ observed for control devices. Deep energy‐level defects are typically associated with intrinsic point defects on the grain boundaries and surfaces of perovskite, such as lead interstitial and substitutional defects.^[^ ^60^ ^]^ Therefore, the variation of deep‐level defects could be attributed to the tight binding of SID to Pb—I and SnO_2_ film surfaces, suppressing the generation of nonradiative recombination centers.

The Mott–Schottky plots of the devices were further investigated, as shown in Figure 3G. The built‐in potentials of the control, SID–SnO_2_, and SID–PbI_2_ devices were found to be 0.85, 0.87, and 0.90 V, respectively, which correlated with the open‐circuit voltage (V_OC_) measured for the subsequent devices. To gain insight into the non‐radiative recombination dynamics within the device, we also tested the short‐circuit current variation trend with different light intensities. As demonstrated in Figure 3H, the SID–SnO_2_ devices (1.65 k_B_T/*q*) and SID–PbI_2_ devices (1.32 k_B_T/*q*) exhibited smaller slopes than the control devices (2.10 k_B_T/*q*). This indicates less trap‐assisted Shockley–Read–Hall recombination and confirms that SID effectively suppresses nonradiative recombination both inside the perovskite and at the rear interface. In summary, the effective reduction in deep‐level defect density and optimization of interfacial contact facilitated by SID modification successfully suppressed nonradiative recombination inside the perovskite and at the rear interface. This prevented charge accumulation and ultimately facilitated carrier transport.

### 2.5 Device Performance

**Figure 4:**
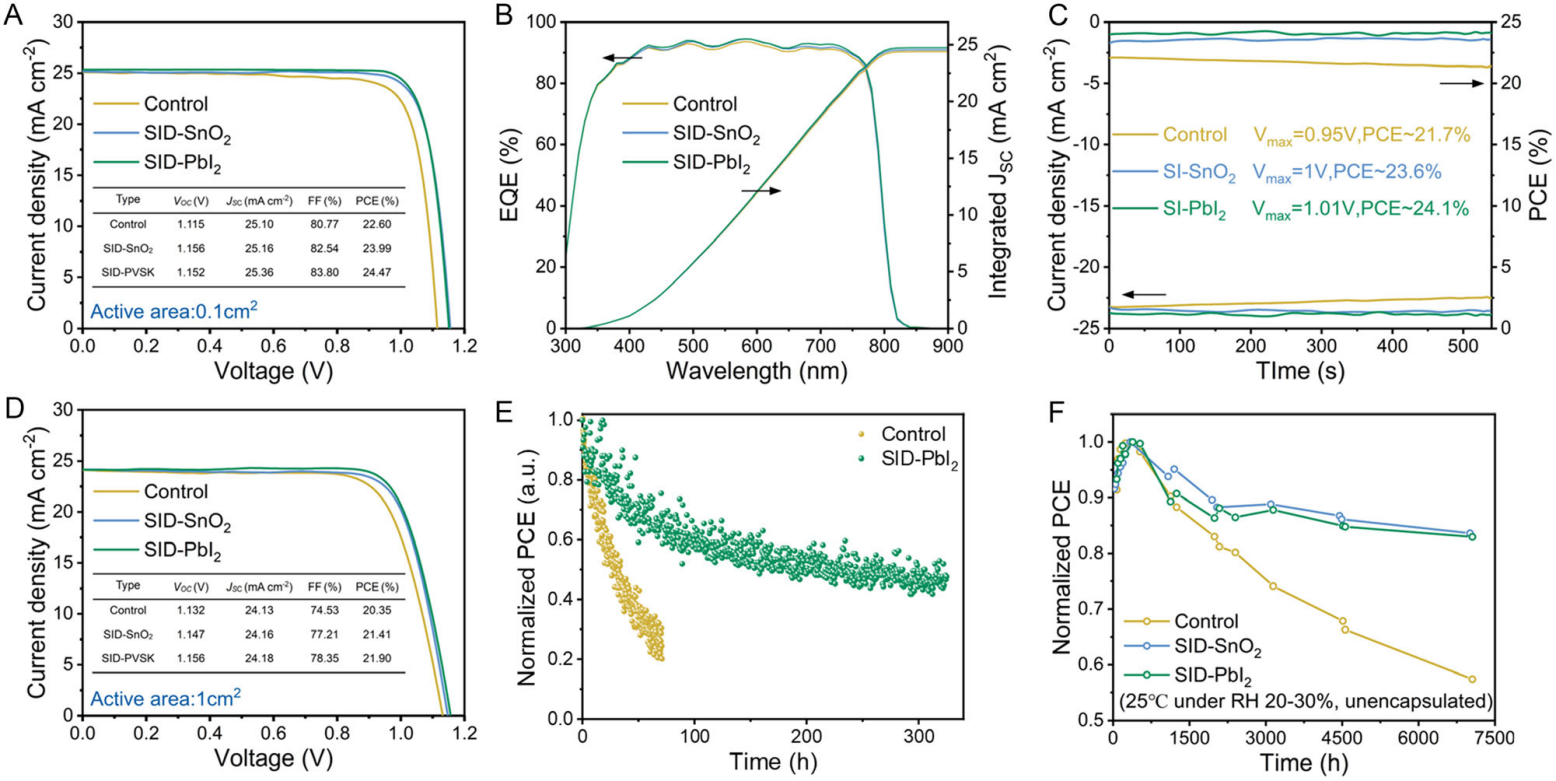
Photovoltaic performance and stability tests of PSCs. A) *J–V* curves and photovoltaic parameters of the 0.1 cm^2^ PSCs. B) The EQE spectra and corresponding integrated *J*_SC_ of the PSCs. C) Stabilized maximum power output at the MPP to obtain the stabilized PCEs. D) *J–V* curves and photovoltaic parameters of the 1 cm^2^ PSCs. E) Operational stability test conducted at MPP for the control and SID–PbI_2_ device. F) Long‐term stability of unencapsulated PSCs in an ambient environment of 20–30% relative humidity at 25 °C.

Further assessment of the photovoltaic performance and stability of the devices was conducted. Figure S21, Supporting Information shows that the optimal treatment concentrations were 1 mg mL^−1^ for SID–SnO_2_ and 2 mg mL^−1^ for SID–PbI_2_. The current density–voltage (*J–V*) curves and corresponding photovoltaic performance parameters of the champion photovoltaic devices for control, SID–SnO_2_, and SID–PbI_2_ under AM 1.5 G illumination (100 mW cm^−2^) are compared in **Figure** **4**A. The control device demonstrated a PCE of 22.59%, with a *V*_OC_ of 1.114 V, short‐circuit current (*J*_SC_) of 25.09 mA cm^−2^, and fill factor (FF) of 80.76%. In contrast, the SID‐PbI_2_ devices showed a significantly enhanced FF value of 83.79%, along with an increased *V*_OC_ of 1.155 V and *J*_SC_ of 25.35 mA cm^−2^, yielding a boosted champion PCE of 24.47% under reverse scan. The SID–SnO_2_ device also significantly increased *V*_OC_ and FF, which were measured to be 1.155 V and 82.53%, respectively. In addition, there was a slight increase in *J*_SC_ of 25.15 mA cm^−2^, resulting in an improved PCE of 23.99% in the reverse scan. The significant enhancement in *V*_OC_ and FF indicated that the two‐site passivation strategy based on SID effectively inhibited nonradiative recombination in the perovskite film and SnO_2_ interface while promoting the separation and extraction of photogenerated carriers simultaneously. Correspondingly, the SID–SnO_2_ and SID–PbI_2_ devices showed a reduced hysteresis index (h) from 5.18% to 3.92% and 4.32%, respectively (Figure S22 and Table S3, Supporting Information). Three types of devices were manufactured under the same conditions, and the statistical analysis confirmed that the devices treated with SID demonstrated improved overall photovoltaic performance parameters, indicating acceptable reproducibility (Figure S23, Supporting Information).

Figure 4B presents the external quantum efficiency (EQE) spectra of the corresponding devices. The generally improved absorption of the modified devices over a wide range of wavelengths is likely attributed to the slight increase in the thickness of the perovskite films, which was confirmed by SEM. The integrated J_SC_ was calculated to be 24.39, 24.55, and 24.73 mA cm^−2^ for the control, SID–SnO_2_, and SID–PbI_2_ devices, respectively, with less than 5% reasonable discrepancy from the *J*_SC_ value obtained from the *J–V* measurements. To assess the actual performance of the PSCs, the stabilized power output was performed to determine the power output stability of the devices at the maximum power point (MPP) (Figure 4C). When biased at 1.00 and 1.01 V, respectively, the SID–SnO_2_ and SID–PbI_2_ devices achieved stabilized PCE of 23.6% and 24.1%, significantly improved compared to 21.7% for the control device at a bias voltage of 0.95 V. Moreover, we fabricated large‐area (1.0 cm^2^) cells using the same process as that for the small devices shown in Figure 4D. As a result, the SID–PbI_2_ devices achieved a champion PCE of 21.90%, demonstrating consistent performance with the 0.1 cm^2^ device (Figure S24 and Table S4, Supporting Information).

Ensuring the long‐term stability of PSCs in various working environments is crucial for their practical application. Environmental factors, such as water, oxygen, and ultraviolet light, can often lead to device degradation and a subsequent reduction in efficiency. The encapsulated SID–PbI_2_ device maintained 47% of its original PCE after 330 h of MPP tracking in the environment. In contrast, the control device showed a degradation to 20% of its original PCE after 70 h (Figure 4E). The enhancement of operational stability may result from the SID passivation effect and the strong combination of SID with PbI_2_, decreasing the PbI_2_ content in perovskite. Next, we tested the long‐term operational stability of unencapsulated devices by monitoring their PCE over time in both nitrogen glove boxes and ambient conditions. As shown in Figure S25, Supporting Information, the control device degraded by over 20% after 7000 h in a nitrogen glove box at 25 °C, while the SID–SnO_2_ and SID–PbI_2_ devices retained 96% and 93% of their initial efficiency during the same period. In order to further evaluate the humidity stability of the devices, we stored the unencapsulated devices in a dry cabinet with a controlled humidity of 20–30% (Figure 4F). After 7000 h, we observed that the SID–SnO_2_ device maintained the highest PCE (retaining 83% of its initial PCE) compared to the other devices. The control and SID–PbI_2_ devices retained 57% and 82% of their initial PCE, respectively. Incidentally, the fluctuating efficiency could perhaps be attributed to irreversible electrochemical history or different oxidation times before testing. Defects at the interface usually provide a pathway for moisture and oxygen from the air to invade the unprotected perovskite film, and ion migration under light can typically destroy the perovskite lattice structure, leading to rapid device degradation. Therefore, SID effectively passivated the defects at the SnO_2_ interface, reducing the binding sites available for moisture and oxygen. In addition, the tight binding of SID to the [PbI_3_]^−^ lattice in the perovskite films also effectively suppressed ion migration.

## 3 Conclusion

In summary, this study presents MLP strategy that utilizes SID to effectively passivate deep‐level defects within the perovskite phase and at the SnO_2_ interface, highlighting the unique properties of SID. The first step involves SID acting as an effective anchor for the PbI_2_ lattice through both Lewis‐base coordination and hydrogen bonding coupling, resulting in reduced Pb–I interstitial and substitutional defects and suppressed nonradiative recombination within the perovskite films. Moreover, the regulation of the crystallization process of PbI_2_ by SID promotes organic cation embedding into the PbI_2_ lattice, resulting in high‐quality films with increased thickness and penetrating grains. The second step involves the auxiliary buried‐interface passivation by the postdeposited film over the SnO_2_ substrate, leading to reduced charge accumulation at the interface and enhanced carrier extraction and transport. The SID–PbI_2_ devices based on a two‐step process achieved an impressive PCE of 24.47%, and the unencapsulated devices maintained 93% and 82% of their initial efficiencies after aging in a nitrogen glove box at 20 °C and 30% RH for more than 7000 h. This study provides valuable insights into developing efficient and simple multifunctional passivation through a single process, which could potentially have significant implications for enhancing the performance of PSCs.

## Conflict of Interest

The authors declare no conflict of interest.

## Supporting information

Supplementary Material

## Data Availability

The data that support the findings of this study are available in the Supporting Information of this article.
