# Supplementary material for: Auxiliary Buried‐Interface Passivation Toward Stable and Low‐Recombination‐Loss Perovskite Photovoltaics
Source: Small Sci. 2023 Nov 27;4(1):2300218. doi: 10.1002/smsc.202300218 (PMC11935047; doi:10.1002/smsc.202300218)
Supplement: Supplementary file 1 — Supplementary Material [file SMSC-4-2300218-s001.pdf]

Supporting Information

Auxiliary Buried-Interface Passivation Toward Stable and Low-Recombination-Loss Perovskite Photovoltaics

Tinghao Li[#], Can Wang[#], Chongzhu Hu, Ni Zhang, Qiu Xiong, Zilong Zhang, Feng Li, Yingyao Zhang, Jihuai Wu and Peng Gao[*]

[#] T.L. and C.W. are equally contributed to this work.

Reagents and materials

SnO_2 colloid precursor (tin (iv) oxide, 15% in H_2O colloidal dispersion) was purchased from Alfa Aesar. Lead iodide (PbI_2, 99.99%) and lead bromide ($PbBr_2$, 99.99%) were bought from TCI, and succinimide (SID, 99%) was purchased from Aladdin. Formamidinium iodide (FAI), methylammonium bromide (MABr), and methylammonium chloride (MACl) were synthesized according to the procedures in previous publications. The 2,2′,7,7′-tetrakis[N,N-di(4-methoxyphenyl)amino]-9,9′-spirobifluorene(Spiro-OMeTAD) were bought from Shenzhen Feiming Co., Ltd. N,N-Dimethylformamide (DMF, anhydrous), dimethyl sulfoxide (DMSO, anhydrous), isopropanol (IPA, anhydrous), chlorobenzene (CB, anhydrous), acetonitrile (anhydrous), 4-tert-butylpyridine (t-BP, 98%), and bis(trifluoromethane)sulfonimide lithium salt (Li-TFSID, 99.95%) were purchased from Sigma-Aldrich. Succinimide (SID, 99%) was purchased from Aladdin

Preparation of Precursor Solutions

The preparation of all precursor solutions required the condition of the N_2 glovebox, high-purity solvents, precise pipette, and balance, except for the SnO_2 solution to be prepared in air. The preparation of perovskite precursor solution is configured in two parts. Firstly, The succinimide was pre-dissolved in DMF at a concentration of 2 mg mL^{-1}, and 1.45 M PbI_2 was dissolved in DMF (2 mg mL^{-1} SID) and anhydrous DMSO at a volume ratio of 9:1 to obtain PbI_2 precursor solution. Then 70mg FAI, 7mg MABr, and 7mg MACl were dissolved in 1 mL isopropanol to obtain an organic amine salt solution. Next, the Spiro-OMeTAD solution was prepared by dissolving 72.3 mg of Spiro-OMeTAD in 1 mL anhydrous chlorobenzene, then 17.5 μL of Li-TFSID solution (520 mg mL^{-1} in acetonitrile) and 28.8 μL of t-BP were added after its dissolution. All of the above solutions need to be stirred overnight after preparation and filtered with a filter prior to use. Besides, 1 mg succinimide dissolved in 1mL water or ethanol, heated at 50°C, and stirred for 30 minutes before use.

Fabrication of the Devices

The perovskite solar cells were fabricated on fluorine-doped tin oxide (FTO, NSG) substrates with a 6 mm blank area etched on top of each substrate. The etched substrates were first cleaned surface with detergent, then immersed in deionized water, acetone, and ethanol in turn for ultrasonic cleaning for each 15 min, followed by drying in a drying cabinet at 70°C for 30 min to remove the residual solvent. Next, the substrates were cleaned with plasma treatment for 10 min before depositing SnO_2 and perovskite film. Next, 80 μL of SnO_2 colloid solution was spin-coated onto FTO substrates at 3000 rpm for 30 s, then annealed at 150 °C for 30 min to deposit SnO_2 films. Then 80μL of succinimide aqueous solution was spin-coated onto the cooled SnO_2 substrate at 3000 rpm for 30 s, then annealed at 60°C for 15 min to complete the modification of the rear interface. For the deposition of the perovskite layer, 45 μL of SID-doped PbI_2 solution was spin-coated onto the SnO_2 in the N_2 glove box at 1500 rpm for 30 s and annealed at 70 °C for 1 min. After PbI_2 had cooled down to room temperature, 50 μL of organic amine salt solution was spin-coated onto the PbI_2 film at 1300 rpm for 30 s, then the film was taken out from the N_2 glove box and annealed at 150 °C for 15 min in ambient air (30%-40% relative humidity). The nitrogen glove box must be re-flushed for a few minutes to remove residual solvent vapors from the previous step before the spin coating of PbI_2, organic amine salt, and Spiro-OMeTAD solution. Next, the hole transport layer was deposited on top of the cooled perovskite layer by 4000 rpm for the 20s using Spiro-OMeTAD solution. Finally, 80 nm of the gold electrode as the back contact was thermally evaporated at 0.05-0.5 Å s^{-1} under 5×10^{-5} Pa, using a shadow mask. The small device size areas were 0.1 cm^2, and the large device size was 1 cm^2.

Characterization

The calculated molecular electronic static potential (ESP) results were obtained with the Multiwfn 3.7 program.[1] Fourier transform infrared (FTIR) spectrum analysis was performed using an FTIR spectrometer (Thermo Scientific Nicolet iS50) with an ATR accessory. The ^{1}H NMR and ^{13}C NMR spectra were performed on the Bruker AVANCE III 500 MHz spectrometer. The X-ray photoelectron spectrum (XPS) was performed using an X-ray photoelectron spectroscopy system (Axis Supra, Shimadzu) with Al Kα X-ray radiation (1486.6 eV) as the X-ray source. Atomic force microscopy (AFM) measurements and Kelvin probe force microscopy (KPFM) were conducted using a Bruker Multimode-8J microscope air mode and ScanAsyst software. The top-view and cross-section scanning electron microscope (SEM) images and energy dispersive X-ray spectroscopy (EDX) were observed by field emission electron microscopy (Apreo S, Thermo Scientific™). The absorption spectra were measured using a UV−vis spectrophotometer (Cary 5000, Agilent). X-ray diffraction (XRD) patterns were measured by Rigaku X-ray diffractometer (Cu Kα radiation, λ=1.5418 Å). Photoluminescence (PL) and time-resolved photoluminescence (TRPL) were carried out with a series of Fluorescence spectrometers (FLS-980). The excitation and emission wavelengths are 480 and 790 nm for the TRPL measurement, respectively. The tr-PL intensity decay spectra were fitted with the bi-exponential decay function: $I(t) = \gamma_0 + A_1 \exp(-t/\tau_1) + A_2 \exp(-t/\tau_2)$. Where γ_0 is the decay constant, A_1 and A_2 are decay amplitudes, τ_1 is the fast interface charge extraction, and τ_2 is the slow trap assisted recombination.[2] The transient photovoltage (TPV), transient photocurrent (TPC), Electrochemical impedance spectroscopy (EIS), thermal admittance spectroscopy (TAS), and Mott-Schottky measurements were carried out using an electrochemical workstation (Zennium Zahner, Germany). Space-charge limited current (SCLC) measurement was performed on a Keithley 2401 source meter ranging from 0V to 5V. The photovoltaic performance was measured on a source meter (Keithley 2401) under AM 1.5G (0.1 W/cm^2) illumination with a solar simulator (Enli Tech), current density-voltage (J-V)

characteristics were recorded by a source meter (Keithley 2401) with 100 mV/s scan speed. The external quantum efficiency (EQE) spectrum was measured by the EQE system (QE-R 3011, Enli) at DC mode. The stability test devices were stored separately and placed in the air for a period of time to oxidize before the test.

Supplemental items

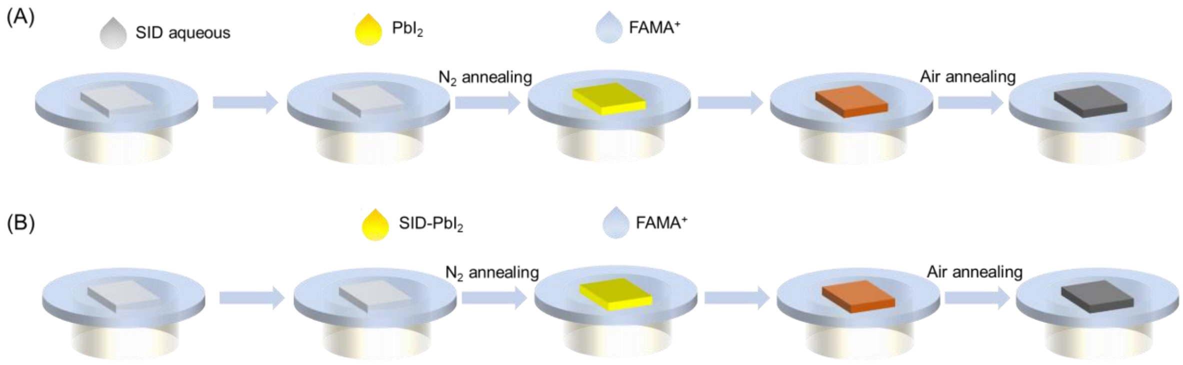

Figure S1. Manufacturing perovskite films.

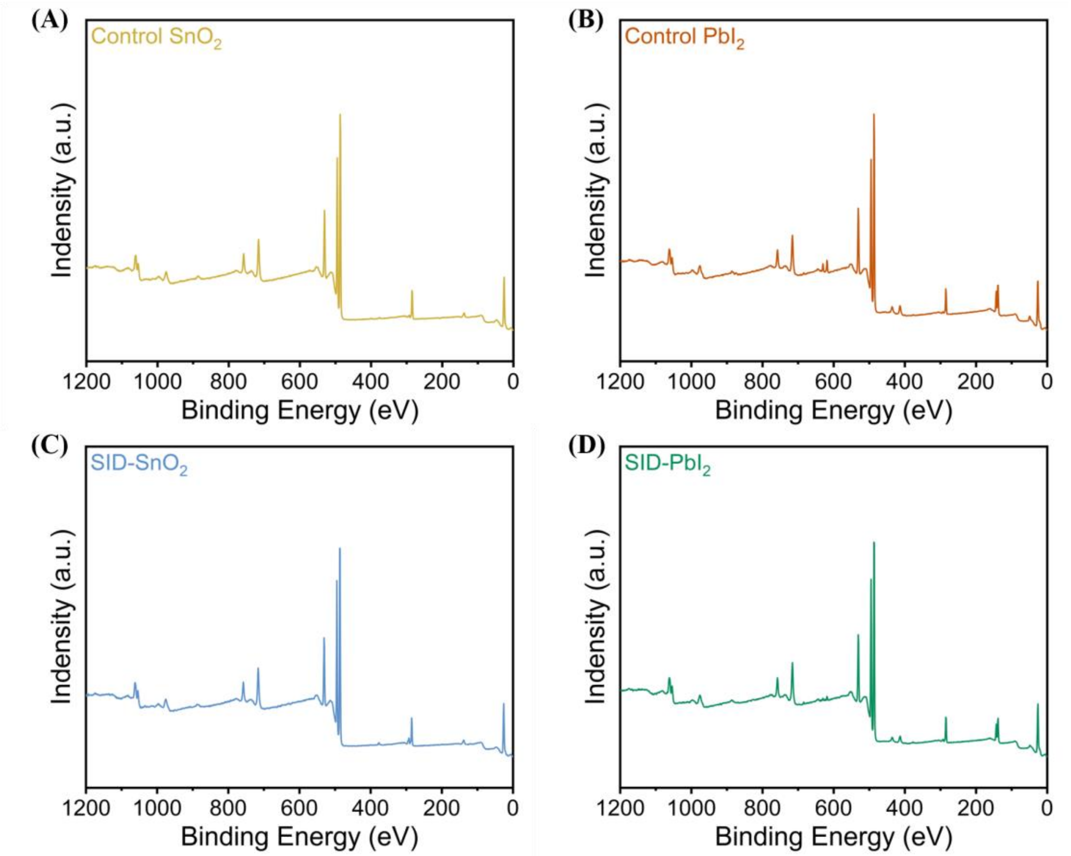

Figure S2. The entire XPS spectra of (A) control SnO_2 film; (B)control PbI_2 modified SnO_2 film; (C) SID-SnO_2 film, and (D) SID-PbI_2 modified SnO_2 film. Note that all films containing PbI_2 were rinsed off with DMSO solvent after annealing to remove the influence of Pb^{2+}.

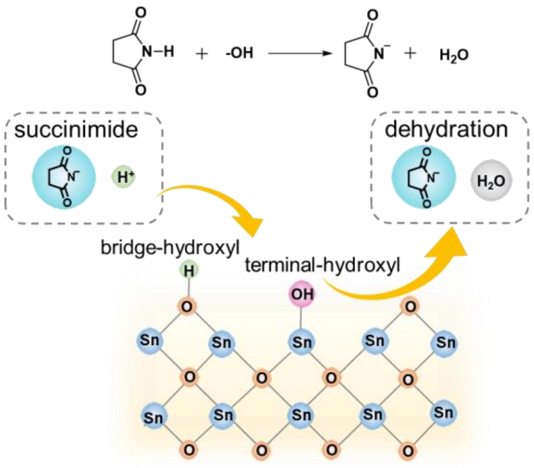

Figure S3. The schematic image of the SID modification on the SnO_2 surface.

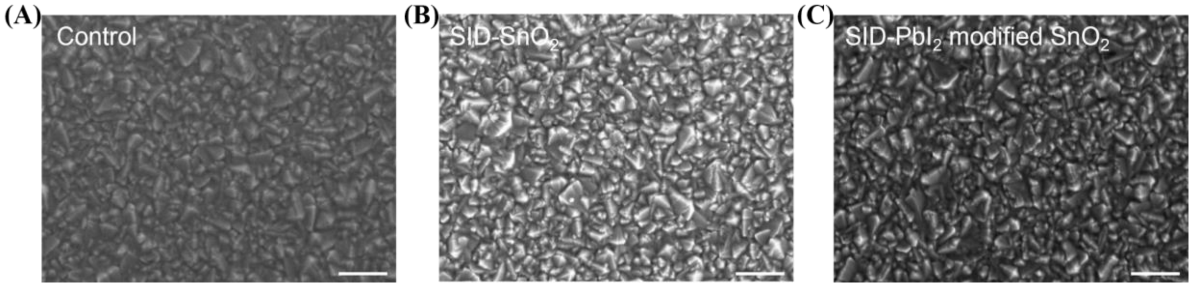

Figure S4. Surface SEM images of (A) control SnO_2 film; (B) SID-SnO_2 film, and (C) SID-PbI_2 modified SnO_2 film. The scale bar is 1 μm.

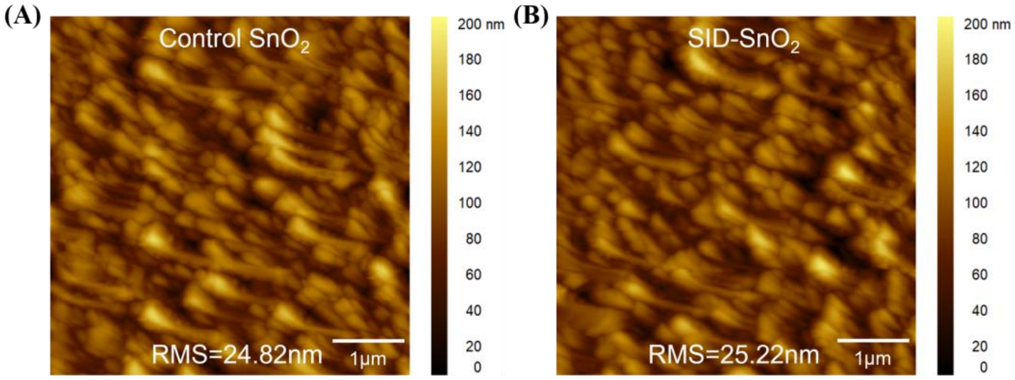

Figure S5. AFM images of SnO_2 films without (A) and with (B) SID modification.

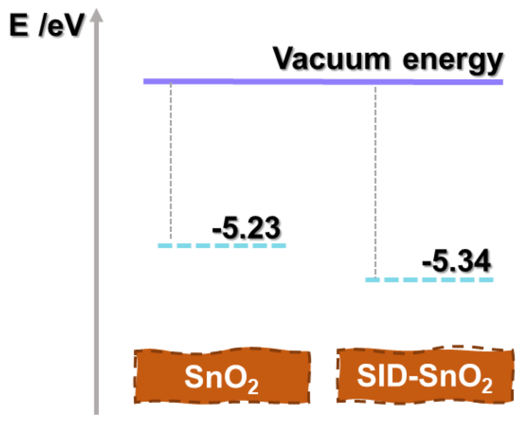

Figure S6. Fermi level of SnO_2 and SID-SnO_2 calculated from CPD.

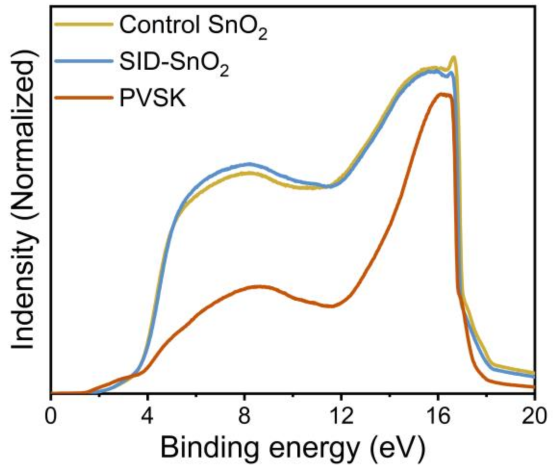

Figure S7. The ultraviolet photoelectron spectroscopy (UPS) of SnO_2 films without and with SID modification and control PVSK film.

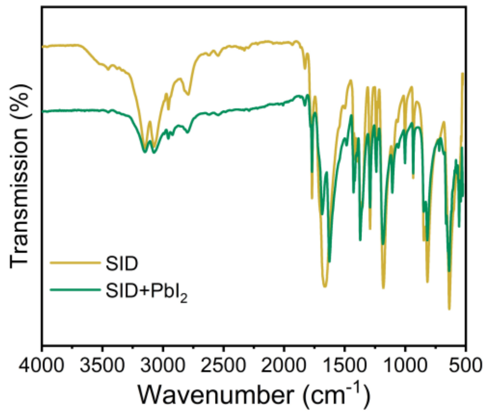

Figure S8. The entire FTIR spectra of SID powder and SID+PbI_2 powder.

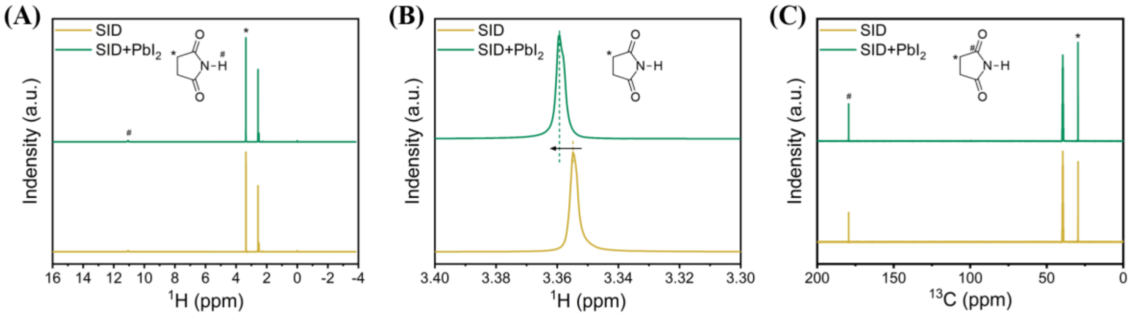

Figure S9. ^{1}H NMR spectra (A,B) and ^{13}C NMR spectra (C).

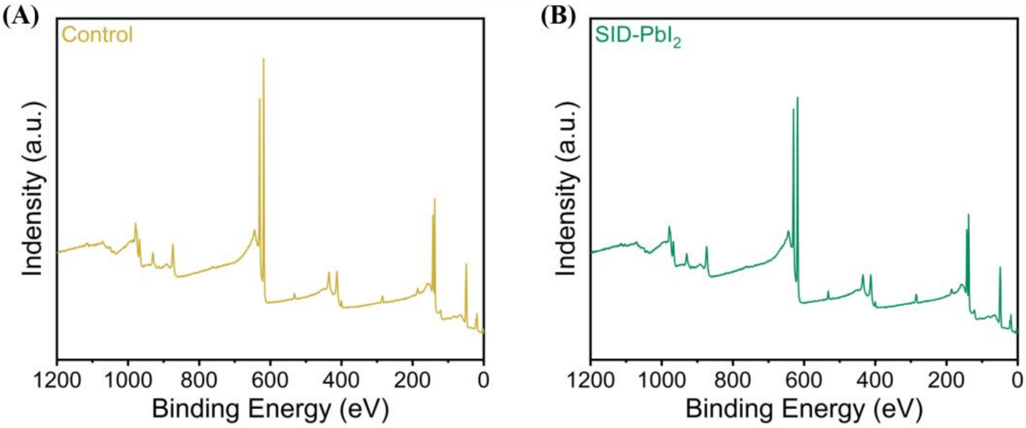

Figure S10. The entire XPS spectra of (A) control perovskite; (B) perovskite with SID additive.

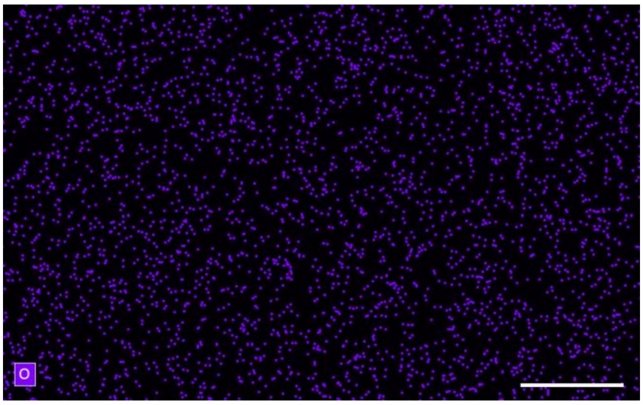

Figure S11. Surface EDS elemental maps of oxygen for perovskite with SID additive. The scale bar is 1 μm.

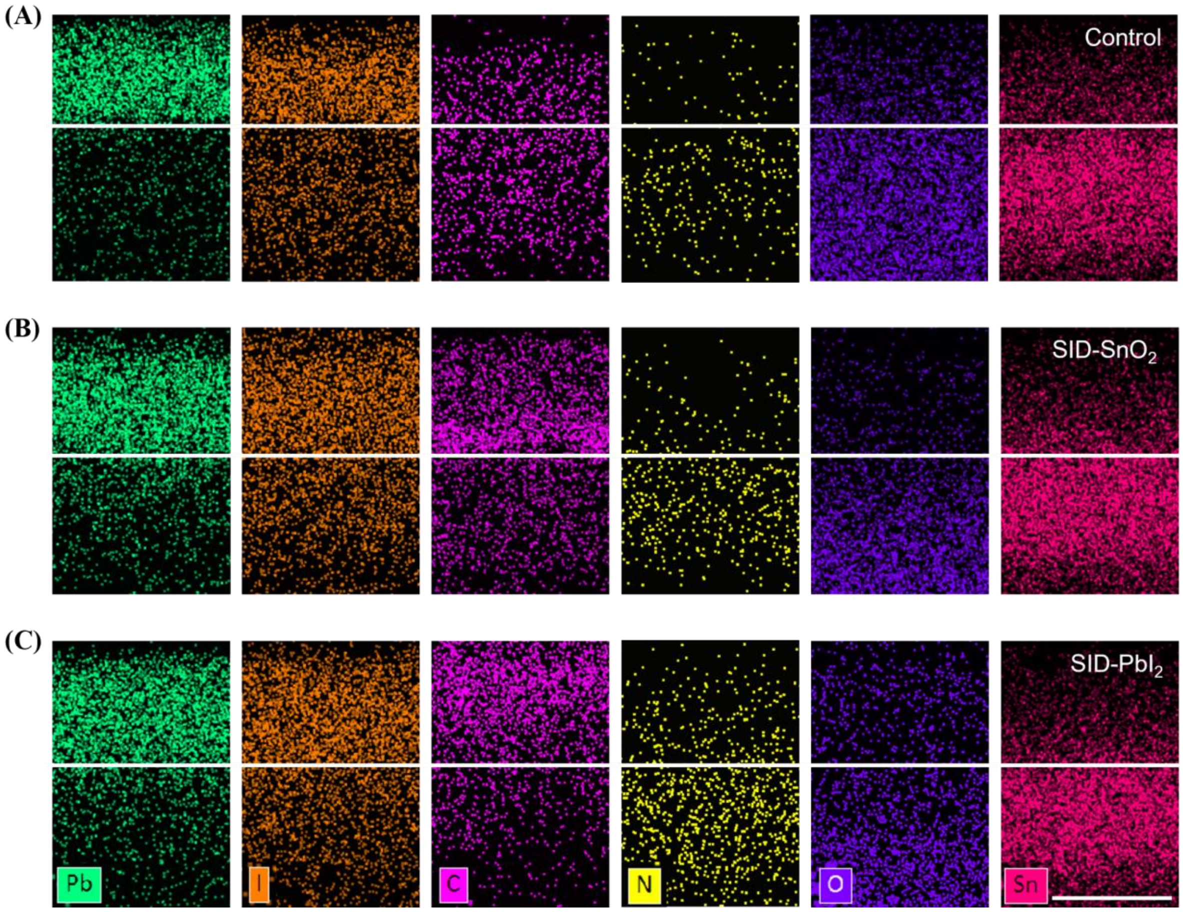

Figure S12. Surface EDS elemental maps of the perovskite film deposited on (A) control SnO_2 substrate; (B) SID-SnO_2 substrate, and (C) perovskite film with SID additive deposited on control SnO_2 substrate. The solid white line represents the approximate interface between the perovskite and SnO_2 film. The scale bar is 500 nm.

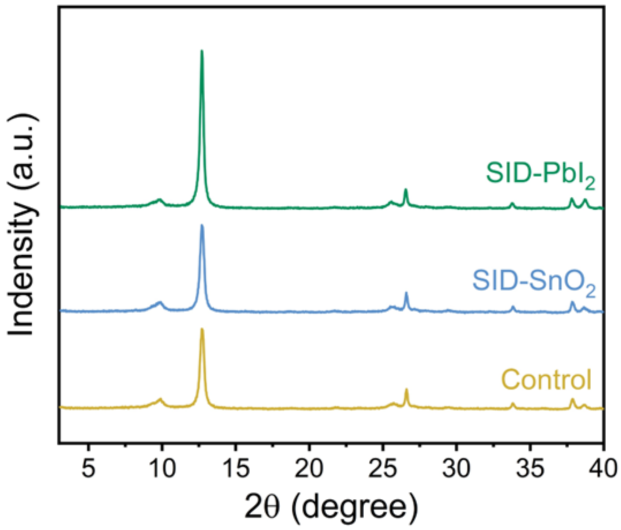

Figure S13. XRD patterns of PbI_2 films with various modifications.

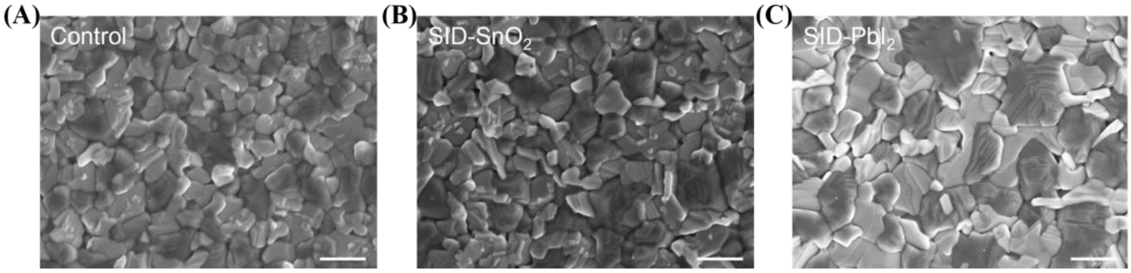

Figure S14. Surface SEM images of the perovskite film deposited on (A) control SnO_2 substrate; (B) SID-SnO_2 substrate, and (C) perovskite film with SID additive deposited on control SnO_2 substrate. The scale bar is 1μm.

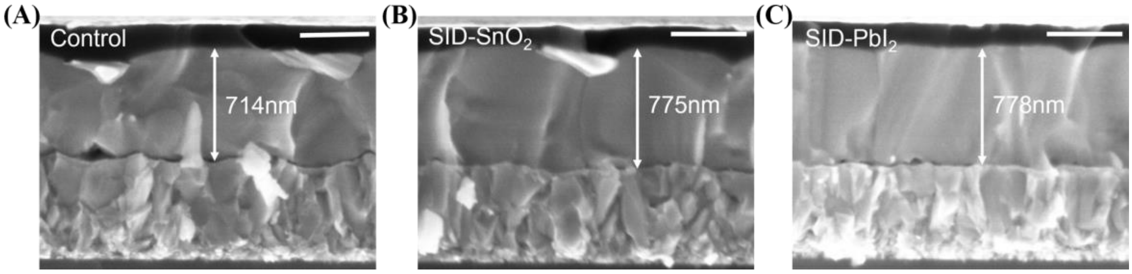

Figure S15. Cross-sectional SEM images of the perovskite film deposited on (A) control SnO_2 substrate; (B) SID-SnO_2 substrate, and (C) perovskite film with SID additive deposited on control SnO_2 substrate. The scale bar is 500nm.

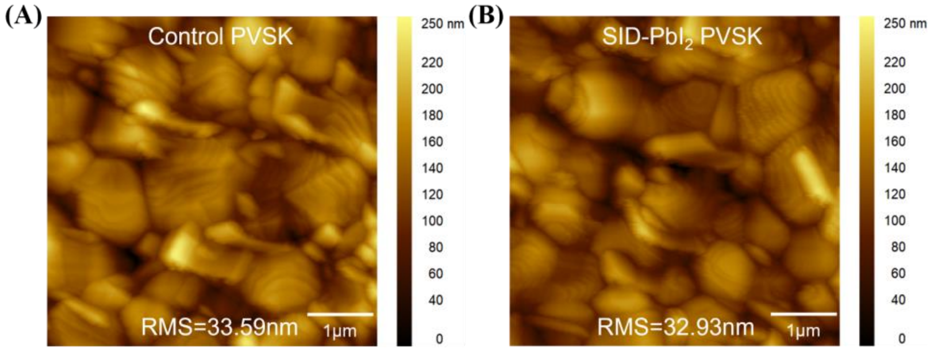

Figure S16. AFM images of perovskite films without (A) and with (B) SID modification.

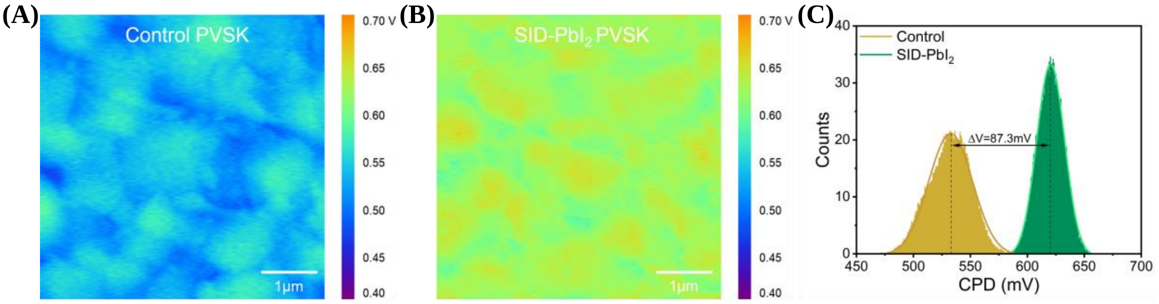

Figure S17. KPFM surface potential distribution of perovskite films without (A) and with (B) SID modification. (C) CPD distributions of perovskite films.

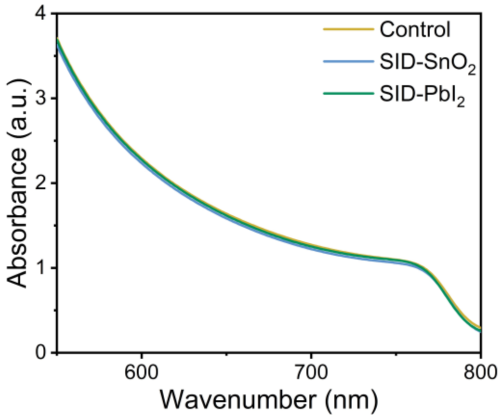

Figure S18. UV-visible absorption of control, SID-SnO_2, and SID-PbI_2 films.

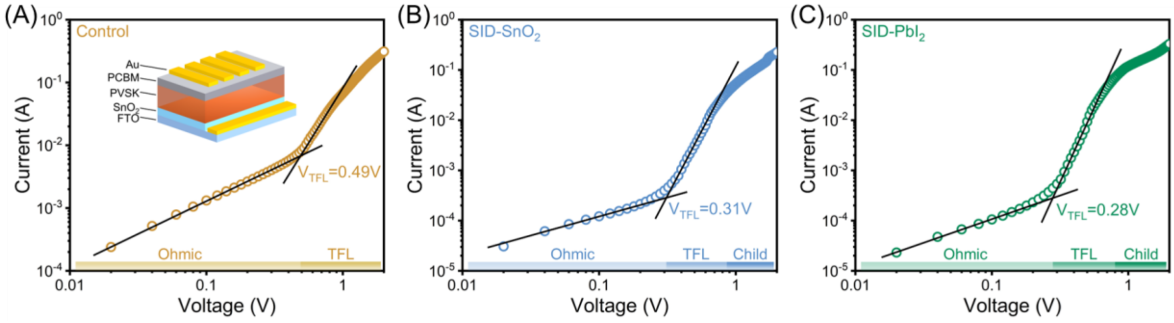

Figure S19. SCLC curves of electron-only devices of (A) control; (B) SID-SnO_2, and (C) SID-PbI_2.

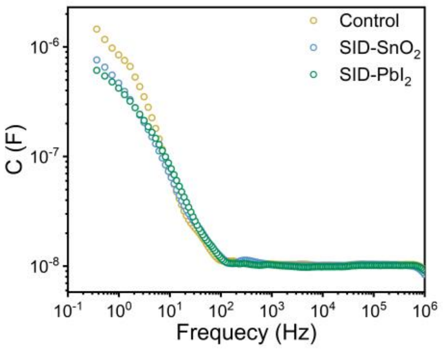

Figure S20. Capacitance–frequency curve.

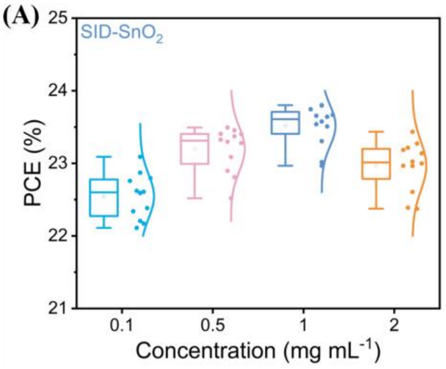

Figure S21. Statistical efficiency of PSCs with (A) SID-SnO_2 and (B) SID-PbI_2 at different concentrations (12 solar cells of each concentration).

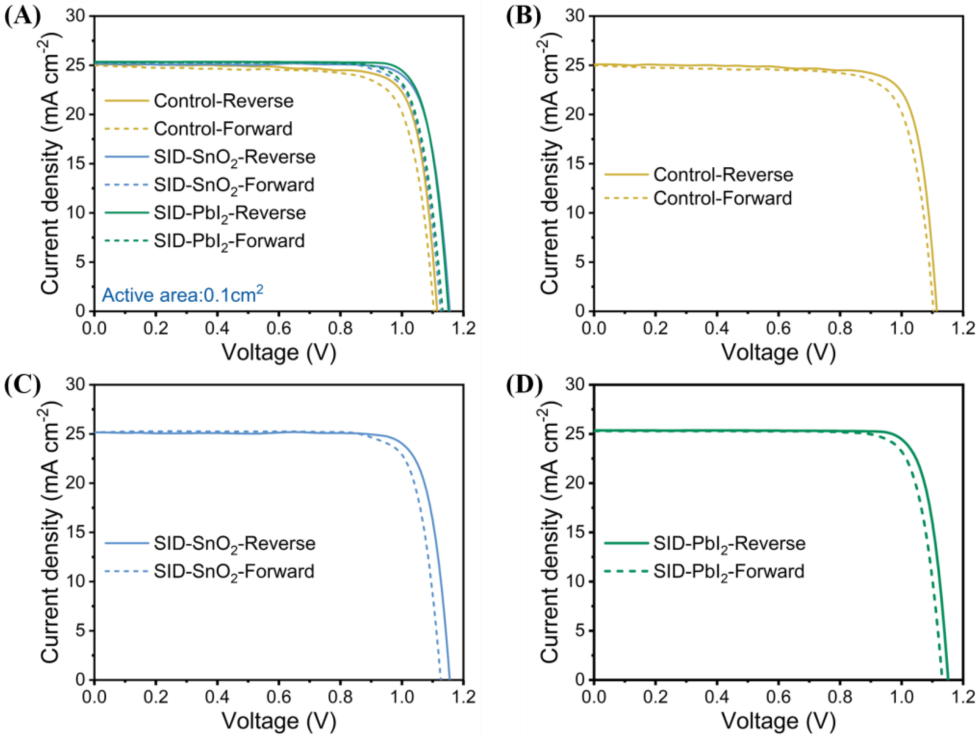

Figure S22. Current-voltage characteristic curves (including forward and backward scan) of the 0.1 cm^2 typical cells of the (A) combined; (B) control; (C) SID-SnO_2, and (D) SID-PbI_2.

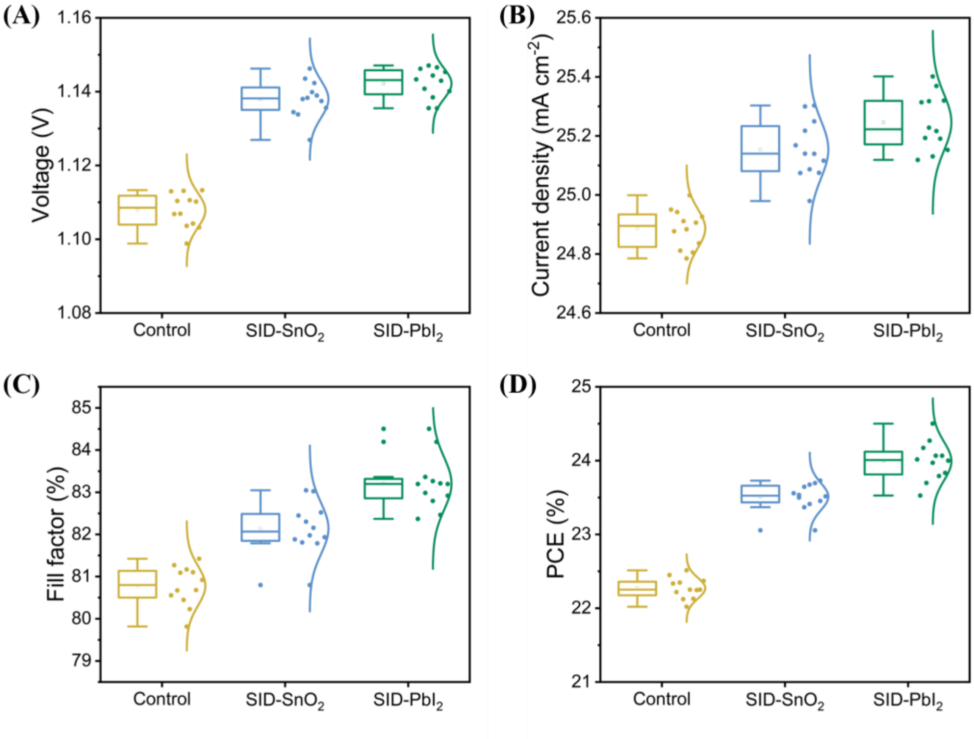

Figure S23. Device performance. Detailed (A) V_{OC}, (B) J_{SC}, (C) FF, and (D) PCE statistical distribution of SID-SnO_2 and SID-PbI_2 PSCs under the optimization of concentration.

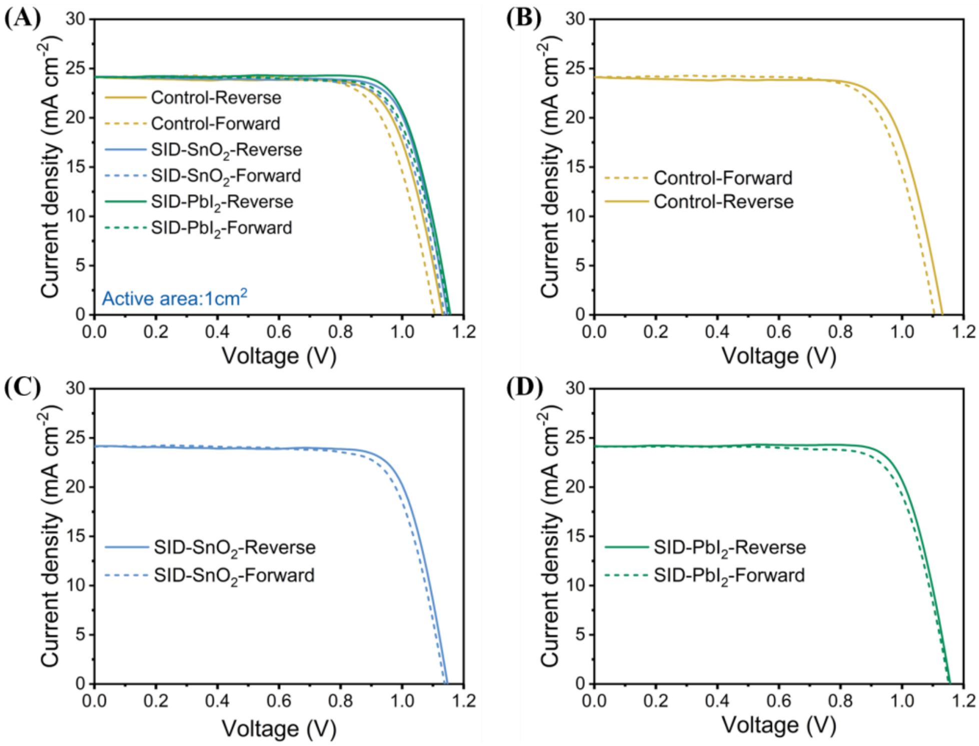

Figure S24. Current-voltage characteristic curves (including forward and backward scan) of the 1 cm^2 typical cells of the (A) total; (B) control; (C) SID-SnO_2, and (D) SID-PbI_2.

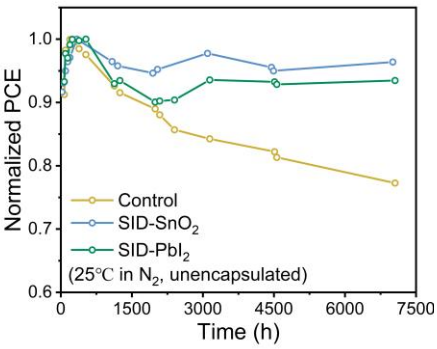

Figure S25. Long-term stability of unencapsulated PSCs in N_2 glove box.

Table S1. The fitted lifetime and parameters deduced from the bi-exponential decay kinetic.

Samples	τ_1 (ns)	A_1	τ_2 (ns)	A_2	τ_{AVE} (ns)
Glass/PVSK	20.41±0.13	0.72±0.002	247.50±1.55	0.23±0.001	201.1
Glass/SID-PVSK	34.89±0.40	0.57±0.003	409.37±2.58	0.33±0.001	361.62
Glass/FTO/ SnO_2/PVSK	6.56±0.04	0.82±0.002	64.03±0.56	0.20±0.001	47.16
Glass/FTO/ SID-SnO_2/ PVSK	5.2±0.02	0.75±0.002	38.04±0.27	0.23±0.001	27.89
Glass/FTO/ SnO_2/SID-PVSK	3.92±0.01	0.81±0.001	29.37±0.21	0.20±0.001	20.49

Table S2. The fitting results of EIS spectra of PSCs.

Samples	R_s (Ω)	R_{ct} (Ω)	R_{rec} (Ω)	C (10^{-9}F)	$CPE\text{-}T$ (10^{-6}F)	$CPE\text{-}P$
Control	10.07	51847	542170	9.37	2.08	0.53
SID-SnO_2	8.382	42114	649370	9.93	2.14	0.69
SID-PbI_2	8.896	48785	677990	9.99	3.03	0.74

Table S3. Photovoltaic parameters of the best-performing PSCs with 0.1cm^2 active area.

Devices	V_{OC} (V)	J_{SC} (mA/cm^2)	FF (%)	PCE (%)	HI
Control RS	1.115	25.10	80.77	22.60	0.052
Control FS	1.104	24.99	77.67	21.42	
SID-SnO_2 RS	1.156	25.16	82.54	23.99	0.039
SID-SnO_2 FS	1.126	25.18	81.29	23.05	
SID-PbI_2 RS	1.152	25.36	83.80	24.47	0.043
SID-PbI_2 FS	1.133	25.29	81.72	23.41	

Table S4. Photovoltaic parameters of the best-performing PSCs with 1cm^2 active area.

Devices	V_{OC} (V)	J_{SC} (mA/cm^2)	FF (%)	PCE (%)	HI
Control RS	1.132	24.13	74.53	20.35	0.041
Control FS	1.106	24.07	73.31	19.51	
SID-SnO_2 RS	1.147	24.16	77.21	21.41	0.039
SID-SnO_2 FS	1.138	24.10	74.99	20.57	
SID-PbI_2 RS	1.156	24.18	78.35	21.90	0.044
SID-PbI_2 FS	1.151	24.14	75.34	20.93	

References

[1] T. Lu, F. Chen, *J. Comput. Chem.* **2012**, *33*, 580.

[2] C. L. Mai, Q. Zhou, Q. Xiong, C. C. Chen, J. Xu, Z. Zhang, H. W. Lee, C. Y. Yeh, P. Gao, *Adv. Funct. Mater.* **2021**, *31*, 2007762.
